# Acknowledgement of Reviewers for 2019

**DOI:** 10.1590/2175-8239-JBN-2020-0001-0017

**Published:** 2020-03-23

The Editor-in-Chief and Associated Editors of the Brazilian Journal of Nephrology express their appreciation to each of the 142 individuals who served as reviewers in 2019, contributing their time, effort and analysis of the manuscripts submitted, to ensure that only the best submitted works are published in journal.

Adriano Ammirati

Alexandre Affonso

Alexandre Cabral

Aline Antunes

Alvaro Silva Filho

Ana Cristina Simões e Silva

Ana Elizabeth Figueiredo

Ana Ravazzani

André Balbi

André Costa

Andrea Carla Bauer

Andrea Stinghen

Andreia Watanabe

Antonio Carlos Seguro

Arman Mushtaq

Bianca Massignan

Bruno Caldin

Bruno Eduardo Balbo

Carlos Aita

Carlos Perez Gomes

Carlucci Ventura

Carmen Tzanno

Carolina Moreira

Cassia Gomes

Cassio Rodrigues

Chris Karohl

Christiane Ramos

Cibele Isaac Saad Rodrigues

Claudia Ribeiro

Cleber Machado de Souza

Clotilde Garcia

Cristian Riella

Cristiane Dias

Cristianne Alexandre

Cristina Martins

Daltro Zunino

Daniel Calazans

Daniela Barreto

Daniela Ponce

Danyelle Rios

Denise Mafra

Dirceu Reis

Domingos Chula

Elizabeth Daher

Elizete Keitel

Emerson Lima

Erika Emy Nishi

Ethiene Macedo

Fabiana Hernandes

Fábio Montenegro

Fellype Barreto

Fernada Santini

Fernanda Gorayeb-Polacchini

Fernando Almeida

Flávio Farias Filho

Francisco Sandro Menezes Rodrigues

Geraldo Silva Junior

Gisele Meinerz

Gustavo Blume

Gyl Silva

Hélady Sanders-Pinheiro

Heloisa Caldas

Ita Heilberg

Itamara Danucalov

James C. Williams

Javier De Arteaga

Jochen Raimann

Joel Veiga

John K. Leypoldt

Jorge Paulo Matos

José Alberto Pedroso

Jose Lopes de Faria

Juliana Mansur

Jyana Morais

Laila Viana

Leandro Lucca

Leda Rabelo

Ligia Pierrotti

Luciana Miyagusku

Lucila Maria Valente

Lúcio Roberto Moura

Luis Arroyo

Luis Fernando Onuchic

Luis Gustavo Modelli de Andrade

Luis Martin

Luiz Antonio Miorin

Manuel Castro

Marcelo Lopes

Márcio Dantas

Marcos de Sousa

Maria Almerinda Ribeiro-Alves

Maria de Fatima Vattimo

Maria Helena Senna

Maria Helena Vaisbich

Marion Deon

Marion Schneider

Marlene Reis

Mauricio Carvalho

Maurilo Leite Júnior

Melani Custodio

Miguel Luís Graciano

Miguel Moysés Neto

Mohammad Hossain

Murilo Guedes

Natália Maria Fernandes

Nilzete Bresolin

Octavio Salgado

Patricia Dalzoto

Paula Hagemann

Paula Orlandi

Paulo H. N. Saldiva

Paulo Rocha

Paulo Suassuna

Precil Neves

Rashad Barsoum

Hugh Rayner

Regina El Dib

Reinaldo Martinelli

Renato Foresto

Renato Watanabe

Ricardo Franco

Roberto Manfro

Roberto Pecoits-Filho

Rodrigo Campos

Rodrigo de Oliveira

Rodrigo Hagemannn

Rogerio Paula

Rosilene Elias

Rui Barros

Samirah Gomes

Sarah Leite

Sérgio Bucharles

Silvia Ribeiro

Stanley Araujo

Tainá Sandes-Freitas

Thais Fernandes

Vanessa Banin

Viktoria Woronik

Vinicius Delfino

Vitor Radunz

Viviane Calice-Silva

Washington Santos

